# Supplementary material for: Multiple mediation effect of coping styles and self-esteem in the relationship between spousal support and pregnancy stress of married immigrant pregnant women
Source: PeerJ. 2023 Oct 24;11:e16295. doi: 10.7717/peerj.16295 (PMC10629385; doi:10.7717/peerj.16295)
Supplement: Supplemental Information 2 [file peerj-11-16295-s002.pdf]

No. ______________

Consent Information

How are you?

My name is Sohyun Moon, and I am majoring in nursing at Chosun University Medical School.

This study is conducted to identify the stress influencing factors of married migrant pregnant women and use them as basic data for the development of pregnancy stress intervention programs.

There is no possibility of physical or mental risk in this questionnaire, and the contents of the questionnaire that you disagree with or withdraw from the questionnaire and respond to will be confidential under an anonymous name, and the time required for the questionnaire is expected to be 20 to 30 minutes.

Data is prohibited from being read only by officials during the study period, and the authority to read is limited to this researcher, one advisor, and if necessary, the judge, and it is discarded after use. It will be a valuable material for this study, and I would appreciate it if you could honestly answer your usual thoughts so that it can be accurate.

Thank you for your cooperation in the research despite your busy schedule. We will give you a small prize as a thank you gift.

Date: July,__________ 2019

Researcher: Department of Nursing, Chosun University Medical School
H.P:010-8622-4509,
E-mail: shmoon@chosun.ac.kr

Consent to participate

- I understood the purpose of this study.
- I refuse to participate in the study at any time during the study period You can abort.
- I agree to participate in the data collection of this study according to my free will.

Names of Participants: (Signature)

Date:

1. Please think of your usual thoughts and choose one of the most similar states and mark O.

Self-esteem		not at all			always
selfesteem1	I feel at much as valuable as other people	1	2	3	4
se2	I feel that I have some good characteristics	1	2	3	4
se3	I feel unsuccessful	4	3	2	1
se4	I feel that I can do things as other people do	1	2	3	4
se5	I feel that I do not have many things to be proud of	4	3	2	1
se6	I have a positive attitude towards myself	1	2	3	4
se7	Generally, I am pleased with myself	1	2	3	4
se8	I would like to have more self-respect	1	2	3	4
se9	Sometimes I feel useless	4	3	2	1
se10	Sometimes I think I am worthless	4	3	2	1
selfesteem11	I feel that I need somone to control me	4	3	2	1

2. How good is your Korean?
   Please mark O where applicable for each of the following questions.

	Never	not good	moderate	Good	Very good
KoreanSpeaking	1	2	3	4	5
KoreanListening	1	2	3	4	5
KoreanReading	1	2	3	4	5
KoreanWriting	1	2	3	4	5

3. I want to know how you usually deal with stress.

Please indicate O where appropriate what method you used to deal with the most recent stress.

Coping strategies		Item	strongly disagree			strongly agree
Problem based coping	stresscopingproblem1	Talked with spouse or other relative about the problem	1	2	3	4
	stresscopingp2	Talked with professional person (e.g., doctor)	1	2	3	4
	stresscopingp3	Tried to find out more about the situation	1	2	3	4
	stresscopingp4	Considered several alternatives for handling the problem	1	2	3	4
	stresscopingp5	Took some positive action	1	2	3	4
	stresscopingp6	Talked with close friends about the worries.	1	2	3	4
	stresscopingp7	Think only about the current state and don't worry about what will happen in the future	1	2	3	4
	stresscopingp8	Drew on my past experiences; I was in a similar situation before	1	2	3	4
Emotional based coping	stresscopinge9	Prayed for a Safe birth	1	2	3	4
	stresscopinge10	Tried to see positive side	1	2	3	4
	stresscopinge11	Tried to step back from the situtation and be more objective	1	2	3	4
	stresscopinge12	Kept my feelings to myself and tried to solve it.	1	2	3	4
	stresscopinge13	Didn't worry about it; figured everything would probably work out fine	1	2	3	4
	stresscopinge14	Sometimes took it out on other people when I felt angry or depressed	1	2	3	4
	stresscopinge15	Tried to reduce the tension by doing what you love, such as cross-stitch, reading, or listening to music.	1	2	3	4
	stresscopinge16	Prepared for the worst	1	2	3	4
	stresscopinge17	I try to forget the problem.	1	2	3	4
	stresscopinge18	Tried to reduce the tension by eating more	1	2	3	4

4. It's about how much help you get from your husband during this pregnancy. Please circle (O) the number that matches your opinion.

social support_spouse supprt	Item	Very dissatisfied					Very satisfied
socialsupportp1	He shares similar experiences with me	1	2	3	4	5	6
socialsupportp2	He helps me keep my morale high	1	2	3	4	5	6
socialsupportp3	He helps me when I need or when I am in trouble	1	2	3	4	5	6
socialsupportp4	He is interested in my daily routine and problems	1	2	3	4	5	6
socialsupportp5	He tries to focus on me when he does something for me	1	2	3	4	5	6
socialsupportp6	He spares time to talk to me about personal and private issue	1	2	3	4	5	6
socialsupportp7	He appreciates the things I do for him	1	2	3	4	5	6
socialsupportp8	He tolerates my ups and downs and unusual behaviors	1	2	3	4	5	6
socialsupportp9	He takes me serious when I am concerned about something	1	2	3	4	5	6
socialsupportp10	He clarifies my condition so that I can understand more easily	1	2	3	4	5	6
socialsupportp11	I know he will be with me when I need help	1	2	3	4	5	6

5. To what extent are the following currently a stress or hastle for you?

pregnancy stress		not stressful at all			very stressful
pregnancystress1	Finantial worries	1	2	3	4
pregnancystress2	Family problems (e.g.children, etc)				
pregnancystress3	Parents-in-law or relatives problems				
pregnancystress4	Sexual life problems				
pregnancystress5	marital relationship problems				
pregnancystress6	Housing or Surrounding environment(including moving) problems				
pregnancystress7	Being exposed to violence (emotional)				
pregnancystress8	Being exposed to violence (physical)				
pregnancystress9	Having lost someone you love recently (e.g., death, divorce, being away from each other)				
pregnancystress10	Problems about consuming alcohol or cigaretts and coffee				
pregnancystress11	Problems about work life(housework or job)				

♣ General characteristics

This is to learn about your general characteristics. Please mark where appropriate.

1.[mother age_mothage]Your age: ( ) years old

[partnet age_partage]Husband's age: ( ) years old

2. [nation]What is your nationality before entering the country?

1. Mongolia / 2. Vietnam / 3. Japan / 4. China / 5.Cambodia / 6.Phillippines

3. [redidura]How long have you lived in Korea?

① Less than 6 months
② 6 months or more to less than 1 year
③ More than 1 to less than 3 years
④ 3 years or more to less than 5 years
⑤ 5 years or more to less than 6 years
⑥ more than six years

4. [edu]What is your level of education?

① No academic background
② elementary school graduate
③ a junior high school graduate
④ a high school graduate
⑤ College graduates or higher

5. [withpartner]Do you currently live with your spouse?

① Yes
② No

6. [job]Do you have a job?

① Yes
② No

7. [partjob]What is your spouse's job?

① a production position
② a skilled commercial engineer
③ General clerical work (office, civil service, teacher)
④ self-employed business
⑤ agricultural and livestock industries
⑥ Other

8. [income]What is your average monthly income?

① less than one million won

② 1 million won or more and less than 1.5 million won
③ 1.5 million won ∽ less than 2 million won
④ Over 2 million won
⑤I don't know

9. [partconv]How much do you talk to your spouse on average per day?

① We don't talk at all.
② Less than 30 minutes
③ 30 minutes to less than an hour
④ Less than 1-2 hours
⑤ More than 2 hours

♣ obstetric characteristics

Please indicate or respond where appropriate

1. [GA]Pregnancy week? (   ) week

2. [pregnancynumber]How many pregnancies are you currently pregnant with? (   )

3. [pregnancyhope]What do you want to get pregnant?

① desired pregnancy
② an unwanted pregnancy

4. [pregnancycompli]Are there any pregnancy-related complications (pregnant hypertension, gestational diabetes) currently in pregnancy?

① Yes
② No

5. [presentbabynumber]How many children do you have now?

① 1
② 2
③ 3
④ 4 or more people

Thank you for your efforts.
